# Supplementary material for: A comparative study of life skills, lifestyle habits and academic performance in health promoting and non-health promoting schools in the Autonomous Community of Aragon, Spain
Source: PeerJ. 2022 Mar 4;10:e13041. doi: 10.7717/peerj.13041 (PMC8900614; doi:10.7717/peerj.13041)
Supplement: Supplemental Information 1 [file peerj-10-13041-s001.pdf]

SOCIODEMOGRAPHIC VARIABLES

1. Are you a boy or a girl?

□ Boy □ Girl

2. What grade are you in?

3. Date of birth

day-month-year

4. How many brothers or sisters do you have?

□ None □ 1 □ 2 □ 3 □ + of 3

5. If you have siblings, where do you rank among them?

□ Oldest □ Middle □ Youngest □ Middle □ Youngest

6. All families are different and we want to know what yours is like. Answer by thinking about the house where

you live all or most of the time and point out the people who live there.

6.1. Adults

□ Mother

□ Father

□ Father's partner

□ Mother's partner

□ Grandmother

□ Grandfather

□ Foster parent

□ Juvenile facility or residence

□ Other adult

Children: How many siblings live in this household?

Include step, foster or adoptive siblings if you have them.

foster or adoptive siblings if you have them

□ Siblings

□ Sisters

7. Father's level of education

□ No education □ Primary school □ Secondary school □ Vocational education (tecnich)

□ University studies

8. Mother's level of education

□ No education □ Primary school □ Secondary school □ Vocational education (tecnich)

□ University studies

9. How much do you weigh approximately?

kg

10. How tall are you approximately?

cm

11. Would you say your health is:

□ Excellent □ Good □ Fair □ Poor □ Fair □ Poor

LIFE SKILLS

1. COGNITIVE SKILLS

Baessler and Schwarzer's General Self-Efficacy Scale.

Rate each of the questions from 1 to 4 according to the following indications:

1 / Incorrect 2 / Barely true 3 / Rather true 4 / True.

1. I can find a way to get what I want even if someone else opposes me.
2. I can solve difficult problems if I try hard enough
3. It is easy for me to persist in what I have set out to do until I reach my goals.
4. I am confident that I could effectively handle unexpected events
5. Thanks to my qualities and resources I am able to overcome unforeseen situations.
6. When I find myself in difficulties, I can remain calm because I have the necessary skills to handle difficult situations
7. Whatever comes my way, I am usually able to handle it.
8. I can solve most problems if I try hard enough.
9. If I find myself in a difficult situation, I can usually figure out what I should do.
10. When faced with a problem, I can usually come up with several alternatives for how to solve it.

2. SOCIAL SKILLS

Social skills assessment checklist (Goldstein et. al. 1980) (Early social skills and advanced social skills).

Rate each of the questions from 1 to 5 on how well you use the skill according to the following indications:

1 / Never 2 / Very seldom 3 / Sometime 4 / Often 5 / Always.

1. Do you pay attention to the person who is speaking to you and make an effort to understand what they are saying?

2. Do you start a conversation with other people and then can you keep it going for a moment?

3. Do you talk to other people about things that interest you both?

4. Do you choose the information you need to know and ask the right person for it?

5. Do you tell others that you are grateful to them for something they have done for you?

Did they do for you?

6. Do you make an effort to meet new people on your own initiative?

7. Do you introduce new people to others?

8. Do you tell others what you like about them or what they do?

9. Do you ask for help when you need it?

10. Do you join a group to participate in a certain activity?

11. Do you clearly explain to others how to do a specific task?

12. Do you pay attention to instructions, ask for explanations, and carry out instructions correctly?

13. Do you apologize to others when you have done something you know is wrong?

14. Do you try to persuade others that your ideas are better and will be more useful than those of others?

3. SKILLS RELATED TO AFFECT MANAGEMENT

Affective Balance Scale

Rate each of the questions from 1 to 3 according to the following indications:

1 / Little or never 2 / Sometimes 3 / Usually or a lot.

1. Have you ever felt annoyed by someone?

2. Have you ever felt very lonely or distant from people?

3. Did you feel that things were going the way you wanted them to go?

4. Did you feel worried a lot?

5. Have you ever felt happy to have good friends?

6. Have you been afraid of what might happen?

7. Have you felt particularly stimulated or interested in anything?

8. Have you ever felt depressed or very unhappy?

9. Have you felt full of energy?

10. Did you feel very tired?

11. Did you feel very nervous, overwhelmed, or tense?

12. Did you feel like you were having a lot of fun?

13. Did you feel very cheerful or happy?

14. Did you feel like crying?

15. Did you feel euphoric (very happy or joyful)?

16. Did you feel confident about the future?

17. Did you feel bored?

18. Did you feel happy or satisfied for having achieved something?

HABITS

1. EATING

1.1. How often do you usually eat breakfast on days you have to go to school or high school?

□ Never □ 1 day □ 2 days □ 3 days □ 4 days □ 5 days □ 1 day □ 2 days □ 3 days □ 4 days □ 5 days

1.2. How often do you usually eat breakfast on weekend days?

□ Never □ 1 day □ 2 days □ 1 day □ 2 days

1.3. How many times a week do you usually eat or drink...?

2. SLEEP

2.1. How many hours do you usually sleep at night during the week?

□ - 5 h. □ 5 - 7 h. □ 7 - 9 h. □ + 9

2.2. What time do you usually go to bed when you have school or high school the next day?

□ Before 9 p.m. □ 9 - 10 p.m. □ + 9 □ 21 - 22 h □ 22 - 23 h □ 23 - 00 h □ + late from

00 h

Fruits

□ Never □ - than 1 time □ 1 time □ 2 - 4 times □ 5 - 6 times □ 1 time a day □ + than 1 time

per day

Potato chips or salty

□ Never □ - 1 time □ 1 time □ 2 - 4 times □ 5 - 6 times □ 1 time a day □ + than 1 time a day □ + than 1 time a day

per day

Vegetables

□ Never □ - 1 time □ 1 time □ 2 - 4 times □ 5 - 6 times □ 1 time a day □ + 1 time a day □ + than 1 time a day

per day

Sweets

□ Never □ - 1 time □ 1 time □ 2 - 4 times □ 5 - 6 times □ 1 time a day □ + than 1 time a day □ + than 1 time a day □ + than 1 time a day

per day

Soft drinks / sugar-sweetened beverages drinks

□ Never □ - 1 time □ 1 time □ 2 - 4 times □ 5 - 6 times □ 1 time a day □ + 1 time a day □ + than 1 time a day

per day

Meat

□ Never □ - 1 time □ 1 time □ 2 - 4 times □ 5 - 6 times □ 1 time daily □ + than 1 time

per day

Fish

□ Never □ - 1 time □ 1 time □ 2 - 4 times □ 5 - 6 times □ 1 time per day □ + than 1 time per day □ + 1 time per day

per day

Milk or dairy

□ Never □ - 1 time □ 1 time □ 2 - 4 times □ 5 - 6 times □ 1 time a day □ + 1 time a day □ + than 1 time a day

per day

Cereals

□ Never □ - 1 time □ 1 time □ 2 - 4 times □ 5 - 6 times □ 1 time daily □ + than 1 time

per day

What time do you usually go to bed on weekends and during vacations?

□ Before 10 p.m. □ 22 - 23 h □ 23 - 00 h □ 00 - 2 h □ 2 - 4 h □ + late from 4 h

3. PHYSICAL ACTIVITY

3.1. Outside of school hours: How often do you engage in any physical activity in your free time that causes you to sweat or become short of breath?

□ Everyday

□ 4 o 6 times a week?

□ 2 o 3 times per week

□ 1 time per week

□ 1 time per month

□ – from 1 time per month

□ Never

3.2. Outside school hours, how many hours a week do you usually engage in any physical activity that cause you to become sweaty or short of breath during your free time?

□ None □ 30 minuts approx. □ 1 h approx.□ 2 - 3 h approx. □ 4 - 6 h □ + than 6 h

3.3. Which of the following types of activities do you usually practice in your free time?

Please tick one box for each line.

Physical activities in team activities (soccer, basketball...)

□ Never □ Rarely (2 – 3 times a month) □ 1 time per week □ 2 – 3 times per week

Individual physical (swimming, running, athletics swimming, athletics, cycling cycling...)

□ Never □ Rarely (2 – 3 times a month) □ 1 time per week □ 2 – 3 times per week

3.4. How do you usually go to school?

□ Walking □ Bicycle □ Car □ Bus □ Bus

4. SCREENS

4.1. How many hours a day, in your free time, do you usually spend playing games on the computer, videoconsol, Tablet, Smartphone or other electronic device (not including motion games or physical exercise)?

Weekdays:

□ None □ 30 min □ 2 h □ 3 - 5 h □ 5 - 7 h □ + than 7 h

Weekend days:

□ None □ 30 min □ 2 h □ 3 - 5 h □ 5 - 7 h □ + de 7 h

4.2. How many hours a day do you usually spend watching television, videos (youtube or similar), movies, series and other entertainment on a screen?

Weekdays:

□ None □ 30 min □ 2 h □ 3 - 5 h □ 5 - 7 h □ + than 7 h.

Weekend days:

□ None □ 30 min □ 2 h □ 3 - 5 h □ 5 - 7 h □ + of 7 h.

4.3. How many hours a day, in your free time, do you usually spend using electronic devices such as computers, tablets or smartphones to do homework, work, surf the Internet or social networks (Facebook, Twitter, Snapchat...)?

Weekdays:

□ None □ 30 min □ 2 h □ 3 - 5 h □ 5 - 7 h □ + de 7 h

Weekend days:

□ None □ 30 min □ 2 h □ 3 - 5 h □ 5 - 7 h □ + de 7 h

5. CONSUMPTION OF INTOXICANTS

5.1. Do any of the following people smoke?

Mother:

□ Daily □ Sometimes □ Does not smoke □ Don't know.

Father:

□ Daily □ Sometimes □ Doesn't smoke □ Don't know

Sibling:

□ Daily □ Sometimes □ Doesn't smoke □ Don't know

Best friend:

□ Daily □ Sometimes □ Doesn't smoke □ Don't know

5.2. How often do you currently smoke tobacco?

□ Everyday □ 1 time per week □ - 1 time per week

5.3 How often do you drink any alcohol? Count even those times when you only drink a small amount

Wine

□ Every day □ Every week □ Every Months □ Rarely □ Never

Liquors drunk alone or with soda- soft drink

□ Every day

□ Every week

□ Every Months

□ Rarely

□ Never

Liquors in the form of Shot glasses

□ Every day

□ Every week

□ Every Months

□ Rarely

□ Never

Other alcohol

□ Every day

□ Every week

□ Every Months

□ Rarely

□ Never

5.4 Have you ever taken one or more of these drugs in your life?

Cocaine

□ Never □ 1 - 2 times □ 3 - 5 times □ 6 - 9 times □ 10 – 19 times □ 20 – 29 times

□ 30 o + times

Hashish or marijuana (joints)

□ Never □ 1 - 2 times □ 3 - 5 times □ 6 - 9 times □ 10 – 19 times □ 20 – 29 times

□ 30 o + times

Extasy,npills, MDMA...

□ Never □ 1 - 2 days □ 3 - 5 days □ 6 - 9 days □ 10 – 19 days □ 20 – 29 days

□ 30 o + days

Amphetamines or speed

□ Never □ 1 - 2 times □ 3 - 5 times □ 6 - 9 times □ 10 – 19 times □ 20 – 29 times

□ 30 o + times

Medications to be placed

Never □ 1 - 2 times □ 3 - 5 times □ 6 - 9 times □ 10 – 19 times □ 20 – 29 times

□ 30 o + times

LSD (acid, tripi, hallucinogenic)

□ Never □ 1 - 2 times □ 3 - 5 times □ 6 - 9 times □ 10 – 19 times □ 20 – 29 times

□ 30 o + times

Glue or solvents

□ Never □ 1 - 2 times □ 3 - 5 times □ 6 - 9 times □ 10 – 19 times □ 20 – 29 times

□ 30 o + times

Other drugs

□ Never □ 1 - 2 times □ 3 - 5 times □ 6 - 9 times □ 10 – 19 times □ 20 – 29 times

□ 30 o + times

If you checked off other drugs, indicate which ones:

5.5. At what age did you first do the following things?

Drinking alcohol (somethingmore than tasting it or take a drink)

□ Never □ 11 years of age or younger □ 12 years of age □ 13 years of age □ 14 years of age or older

Getting drunk

□ Never □ 11 years of age or younger □ 12 years of age □ 13 years of age □ 14 years of age or older

Smoking a cigarette (more than one puff)

□ Never □ 11 years of age or younger □ 12 years of age □ 13 years of age □ 14 years of age or older

Using cannabis

□ Never □ 11 years or less □ 12 years □ 13 years □ 14 years or more

Other drugs. Indicate which ones:

ACADEMIC PERFORMANCE

HIGH SCHOOL STUDENTS

Indicates the average or overall grade in each of the subjects, in case of not taking any of them put NO. To must include the grades of the first and second trimester of the school year.

1. Biology and geology
2. Physics and chemistry
3. Geography and history
4. Spanish language and literatura

5. Mathematics
6. Physical Education
7. Religion or Ethical Values
8. Plastic, visual and audiovisual education
9. Music
10. Technology
11. English
12. French
13. Classical culture
